# Stroke Risk Reduction in Atrial Fibrillation Through Pharmacist Prescribing: A Randomized Clinical Trial

**DOI:** 10.1001/jamanetworkopen.2024.21993

**Published:** 2024-07-24

**Authors:** Roopinder K. Sandhu, Miriam Fradette, Meng Lin, Erik Youngson, Darren Lau, Tammy J. Bungard, Ross T. Tsuyuki, Lisa Dolovich, Jeff S. Healey, Finlay A. McAlister

**Affiliations:** 1 Libin Cardiovascular Institute, University of Calgary, Calgary, Alberta, Canada; 2 Division of Cardiology, University of Alberta, Edmonton, Canada; 3 Canadian VIGOUR Centre, University of Alberta, Edmonton, Canada; 4 Smidt Heart Institute, Cedars-Sinai Medical Center, Los Angeles, California; 5 Alberta Strategy for Patient-Oriented Research, University of Alberta, Edmonton, Canada; 6 Alberta Health Services Provincial Research Data Services, Edmonton, Canada; 7 Division of General Internal Medicine, University of Alberta, Edmonton, Canada; 8 Leslie Dan Faculty of Pharmacy, University of Toronto, Toronto, Ontario, Canada; 9 Population Health Research Institute, McMaster University, Hamilton, Ontario, Canada

## Abstract

**Importance:**

Major gaps in the delivery of appropriate oral anticoagulation therapy (OAC) exist, leaving a large proportion of persons with atrial fibrillation (AF) unnecessarily at risk for stroke and its sequalae.

**Objective:**

To investigate whether pharmacist-led OAC prescription can increase the delivery of stroke risk reduction therapy in individuals with AF.

**Design, Setting, and Participants:**

This prospective, open-label, patient-level randomized clinical trial of early vs delayed pharmacist intervention from January 1, 2019, to December 31, 2022, was performed in 27 community pharmacies in Alberta, Canada. Pharmacists identified patients 65 years or older with 1 additional stroke risk factor and known, untreated AF (OAC nonprescription or OAC suboptimal dosing) or performed screening using a 30-second single-lead electrocardiogram to detect previously unrecognized AF. Patients with undertreated or newly diagnosed AF eligible for OAC therapy were considered to have actionable AF. Data were analyzed from April 3 to November 30, 2023.

**Interventions:**

In the early intervention group, pharmacists prescribed OAC using guideline-based algorithms with follow-up visits at 1 and 3 months. In the delayed intervention group, which served as the usual care control, the primary care physician (PCP) was sent a notification of actionable AF along with a medication list (both enhancement over usual care). After 3 months, patients without OAC optimization in the control group underwent delayed pharmacist intervention.

**Main Outcomes and Measures:**

The primary outcome was the difference in the rate of guideline-concordant OAC use in the 2 groups at 3-month follow-up ascertained by a research pharmacist blinded to treatment allocation.

**Results:**

Eighty patients were enrolled with actionable AF (9 [11.3%] newly diagnosed in 235 individuals screened). The mean (SD) age was 79.7 (7.4) years, and 45 patients (56.3%) were female. The median CHADS_2_ (congestive heart failure, hypertension, age, diabetes, and stroke or transient ischemic attack) score was 2 (IQR, 2-3). Seventy patients completed follow-up. Guideline-concordant OAC use at 3 months occurred in 36 of 39 patients (92.3%) in the early intervention group vs 23 of 41 (56.1%) in the control group (*P* < .001), with an absolute increase of 34% and number needed to treat of 3. Of the 23 patients who received appropriate OAC prescription in the control group, the PCP called the pharmacist for prescribing advice in 6 patients.

**Conclusions and Relevance:**

This randomized clinical trial found that pharmacist OAC prescription is a potentially high-yield opportunity to effectively close gaps in the delivery of stroke risk reduction therapy for AF. Scalability and sustainability of pharmacist OAC prescription will require larger trials demonstrating effectiveness and safety.

**Trial Registration:**

ClinicalTrials.gov Identifier: NCT03126214

## Key Points

**Question:**

Can a pharmacist-led oral anticoagulation (OAC) prescription intervention increase appropriate stroke risk reduction therapy for patients with undiagnosed or undertreated atrial fibrillation (AF) identified in community pharmacies?

**Findings:**

This randomized clinical trial of 80 patients with AF and high stroke risk found a 34% absolute increase in appropriate stroke risk reduction therapy with a pharmacist prescription compared with usual care.

**Meaning:**

Engagement of community pharmacists is a potentially high-yield opportunity to effectively close gaps in the delivery of stroke risk reduction therapy for AF.

## Teaser

This randomized clinical trial compares early and delayed pharmacist intervention for oral anticoagulation therapy among patients with undertreated and newly diagnosed atrial fibrillation in community pharmacies.

## Introduction

Atrial fibrillation (AF) is the most common heart rhythm disorder^1^ and a leading cause of stroke in aging adults.^2,3^ Strokes related to AF are more severe and disabling compared with non-AF strokes.^3,4,5,6,7^ Oral anticoagulation (OAC) therapy is widely available and highly effective for stroke risk reduction and improving survival in AF.^8,9,10,11,12,13^ However, despite accessible clinical guidelines for identifying individuals with AF who stand to benefit from OAC,^14,15,16^ major gaps in the delivery of appropriate OAC therapy persist, leaving a large proportion of persons with AF unnecessarily at risk for stroke and its sequalae. Recent advances in OAC therapeutics offer effective drug choices that are safer and more easily administered and managed than warfarin,^9,10,11,12^ yet large proportions of the population at risk for AF remain undertreated or untreated.^17,18,19,20,21,22,23^ Novel solutions are needed to address persistent gaps in the delivery of OAC therapy for AF.

Growing evidence indicates that pharmacist-based interventions for optimizing cardiovascular disease care, particularly when focused on adherence to guideline-directed therapies, can lead to marked and sustained improvement in clinical outcomes.^24,25,26,27,28,29,30^ The most commonly reported gaps in delivery of OAC therapy include nonprescription, inappropriate medication, or suboptimal dosing,^17,18,19,20,21,22,23^ all of which can be directly and effectively targeted with pharmacist-based interventions.^24,31,32,33,34,35^ The potential for efficacy around AF care is especially promising, as pharmacist-led warfarin anticoagulation clinics have been demonstrated to improve time in the therapeutic range and decrease adverse bleeding events.^36,37,38,39^ Given increasing recognition of both the feasibility and cost-effectiveness of pharmacist-based clinical programs,^40^ several countries have moved toward pharmacist prescribing, either independently or through the use of collaborative practice agreements.^41,42,43,44^

We conducted a patient-level randomized clinical trial (RCT) of early vs delayed pharmacist intervention of OAC therapy for undertreated and newly diagnosed AF in community pharmacies. Secondary objectives assessed patient satisfaction with pharmacist services, clinical events, use of health care services, and OAC therapy adherence at 12 months.

## Methods

This RCT was reviewed and approved by the Health Research Ethics Board at the University of Alberta, and all patients provided written informed consent to participate. The study followed the Consolidated Standards of Reporting Trials (CONSORT) reporting guideline.

### Study Design

The Improving Stroke Risk Reduction in AF Through Pharmacist Prescribing: Program for the Identification of Actionable AF study^45^ was an investigator-initiated, prospective, open-label, RCT of early vs delayed pharmacist intervention of OAC therapy for stroke risk reduction in AF. The study was performed in community pharmacies with at least 1 pharmacist with independent prescribing authority as identified by the Alberta College of Pharmacy and who had access to electronic health records (EHR), including a pharmacy medication database. Pharmacies were invited to participate from an established pharmacy network with prior research experience and others self-identified in response to advertisement in the Alberta Pharmacists’ Association newsletter or by word of mouth among pharmacists. Twenty-seven pharmacies participated in the trial. Pharmacists in all participating pharmacies were provided with a comprehensive online training program consisting of modules reviewing AF epidemiology, stroke risk schema, guideline indications for OAC therapy, OAC type and dosing, and bleeding risk assessment, along with instruction on screening and enrollment processes and research ethics. Further training occurred through telephone and in-person meetings biweekly. Prior to study implementation, a stakeholder team consisting of pharmacists, primary care physicians (PCPs), and community organizations convened to discuss study details and address potential barriers. The trial protocol and statistical analysis plan are provided in Supplement 1. The study was coordinated at the EPICORE Centre, University of Alberta, Edmonton, Canada.

### Patients

Pharmacists identified patients 65 years or older with known, undertreated AF or previously unrecognized AF with 1 additional stroke risk factor (hypertension, type 2 diabetes, heart failure or left ventricular ejection fraction <0.40, or previous stroke or transient ischemic attack confirmed by EHR). Undertreated AF was defined based on established guidelines and conventions for OAC therapy in AF^46,47^ as follows: (1) known AF eligibility for but not receiving OAC therapy (CHADS_2_ [congestive heart failure, hypertension, age, diabetes, and stroke or transient ischemic attack] score ≥2; no OAC contraindications), or (2) known AF and suboptimal dosing (eTable 1 in Supplement 2). Atrial fibrillation was confirmed by either electrocardiographic (ECG [ie, electrocardiogram, Holter, event monitor]) or physician (notes or discharge summary) documentation.

For patients without a history of AF, the pharmacist assessed rhythm by using a mobile ECG apparatus attached to a smartphone or tablet, which allowed a single-lead ECG recording (Kardia; AliveCor) using 2 fingers contacting electrodes for 30 seconds. The single-lead ECG recording includes a highly accurate AF detector (sensitivity, 98% [95% CI, 89%-100%]; specificity, 97% [95% CI, 93%-99%]) approved by the US Food and Drug Administration.^48^ Two ECG recordings were obtained from each patient; if there was disagreement in the interpretation, a third ECG recording was obtained. Physician verification was performed for newly detected AF. For patients with newly diagnosed AF, a 12-lead ECG was required within 24 to 48 hours of the screening session.

Patients with undertreated AF and those with newly diagnosed AF (based on single-lead ECG) eligible for OAC therapy were considered to have actionable AF. Key exclusion criteria included inability to read or understand English, patients considered unreliable concerning requirements for follow-up, or moderate cognitive impairment (≥5 errors on the Short Portable Mental Status questionnaire).^49^ Several recruitment strategies were used, including pharmacists screening of EHR and study participation offered by a telephone invitation and advertisement in local newspapers, senior community associations, and in pharmacies.

### Randomization

Eligible consenting patients were randomized (using block randomization without stratification via a centralized secure website to ensure allocation concealment) in a 1:1 fashion to early vs delayed pharmacist intervention. The study design flow is shown in eFigure 1 in Supplement 2. All PCPs were notified by fax of their patient’s participation in the study and were provided a 1-page study synopsis. The fax also contained information specific to the randomized group.

### Intervention Group

Patients in the early pharmacist intervention group received AF education, blood pressure assessment, and OAC prescription by the pharmacist based on guideline algorithms,^46,47^ with follow-up visits at 1 and 3 months. The fax to the PCP included a summary of current OAC management. The pharmacist notified the PCP regarding any changes to OAC prescription during follow-up visits.

### Control Group

In the delayed intervention group, which served as the usual care control for the first 3 months of the trial, patients received AF education and were encouraged to book a visit for further evaluation with their PCP. A fax was sent to their PCP identifying the patient as having actionable AF and included a current medication list. The fax represents an enhancement over usual care, as such faxes are not usually sent to PCPs after pharmacy visits. After 3 months, patients who did not have OAC optimized by their physician received the same pharmacist intervention as offered at baseline to the early intervention group. Thereafter, all patients were passively followed up to 12 months for assessment of clinical events and OAC adherence.

### Assessments

All study visits were conducted in the community pharmacies. Baseline assessments collected data via patient interview and EHR review (using a provincial electronic medical record system) on demographic characteristics, including race (reported by patients according to categories defined by the investigator as Asian or Pacific Islander; Black or African American; First Nation, Inuit, or Métis; Latin American; South Asian; West Indian; White; other race; or not answered), medical history, health behaviors, anthropometric indexes, medications, laboratory values (complete blood count, basic metabolic panel, liver function), and measured pulse and blood pressure. At follow-up visits, new medical problems, emergency department visits or hospitalizations, embolic events, adherence, bleeding risk assessment, renal function review, drug interactions, blood pressure assessment, and patient education and counseling were completed. Race data were collected owing to health inequities reported in AF.

### Outcomes

The primary outcome was the difference in the rate of guideline-concordant OAC use in the 2 groups at 3 months. We selected a 3-month period for the primary outcome to allow comparison with other quality improvement studies focused on improving OAC use by patients with AF in primary care settings.^50^ The primary outcome was validated by medical record review by a clinical research pharmacist (M.F.) blinded to treatment group allocation.

Patients exposed to the pharmacist-led intervention (early or delayed) were invited to participate in a patient satisfaction survey using a validated Patient Satisfaction With Pharmacist Services questionnaire 2.0 consisting of 22 items related to 3 domains, identified as quality of care, patient-pharmacist relationship, and overall satisfaction using a 4-point, Likert-type scale (1 indicates strongly agree; 2, agree; 3, disagree; and 4, strongly disagree).^51^ For question 19 (“There are some things about my visit with the pharmacist that can be improved”), a higher score was more favorable. Other secondary outcomes included clinical events (bleeding episode, stroke, transient ischemic attack, systematic embolism, acute coronary syndrome, and all-cause mortality), use of health care services (emergency department visit or hospitalization), and OAC therapy adherence (defined as the proportion of prescription days covered ≥80%) at 12 months. Clinical events and use of health care services underwent blinded adjudications by 2 physicians independently of the study.

### Statistical Analysis

Data were analyzed from April 3 to November 30, 2023. In a prior population-based study performed in Alberta, Sandhu et al^52^ found after an initial diagnosis of nonvalvular AF, 49% of patients were prescribed OAC within 3 months. Since there is no baseline rate for prescribing OAC therapy by pharmacists, we surveyed investigators from the Canadian Stroke Prevention Intervention Network to determine a clinically important change in OAC prescribing with intervention. The consensus was that a minimal clinically important difference of 15% (absolute) higher in the intervention group compared with the control group was needed to properly evaluate the intervention; thus our calculated sample size with 1:1 randomization and 80% power with α of .05 was 168 patients per group (370 in total to account for dropouts and loss to follow-up) (see trial protocol and statistical analysis plan in Supplement 1). Unfortunately, with the onset of COVID-19, recruitment was substantially affected and far slower than expected due to 2 provincial lockdowns and a rapid shift to virtual care. Therefore, the decision was taken to close the trial at 80 patients based on logistics and without knowledge of results in either treatment group (which were recorded in blinded fashion by the research pharmacist [M.F.] who was not involved in the decision to close the trial early).

All analyses were performed using the intention-to-treat principle for all randomized patients. Categorical variables are presented as numbers and percentages, and groups were compared using χ^2^ tests. Continuous variables are presented as means and SDs or medians and IQRs, and groups were compared using 2-tailed *t* tests or Wilcoxon rank sum tests for nonparametric distribution. Analyses were performed using SAS, version 9.4 (SAS Institute Inc). Two-sided *P* < .05 indicated statistical significance.

## Results

### Patient Characteristics

From January 1, 2019, to December 31, 2022, we screened 235 patients, 80 of whom underwent randomization at 27 community pharmacies (Figure). Follow-up (in-person or virtual) was complete for 70 patients (87.5%) at 3 months and 63 patients (78.8%) at 1 year (Figure). Among the 80 patients, 9 (11.3%) had newly diagnosed AF and 71 (88.8%) had undertreated AF (5 nonprescription, 66 suboptimal dosing [17 with warfarin and 49 with a direct OAC]). Baseline patient characteristics were balanced between groups (Table). The mean (SD) age was 79.7 (7.4) years; 45 patients (56.3%) were female and 35 (43.8%) were male. In terms of race, 78 patients (97.5%) were White and 2 (2.5%) were other (including First Nation, Inuit, or Métis; Latin American; South Asian; West Indian; not answered; or other). The median CHADS_2_ score was 2 (IQR, 2-3).

**Figure.**
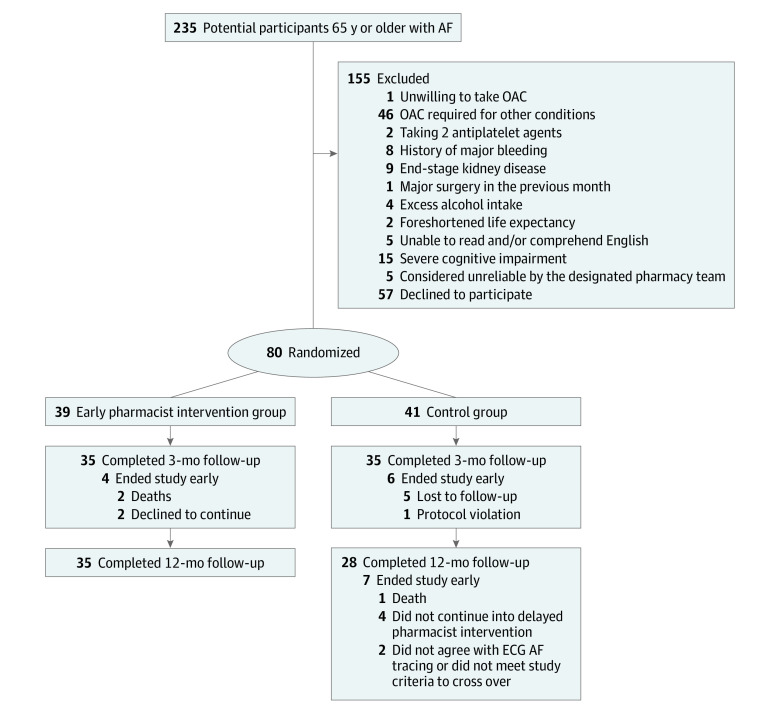
Study Flow Diagram AF indicates atrial fibrillation; ECG, electrocardiogram; OAC, oral anticoagulation.

**Table.** Patient Characteristics

Characteristic	Study group^a^	
	Early pharmacist intervention (n = 39)	Control (n = 41)
Age, mean (SD), y	79.3 (7.6)	80.0 (7.2)
Sex		
Female	22 (56.4)	23 (56.1)
Male	17 (43.6)	18 (43.9)
Race		
White	37 (94.9)	41 (100)
Other^b^	2 (5.1)	0
Baseline systolic BP, mean (SD), mm Hg	120.9 (30.8)	120.6 (36.2)
Baseline diastolic BP, mean (SD), mm Hg	69.6 (22.9)	68.5 (23.4)
Heart rate, mean (SD), bpm	63.2 (22.9)	64.5 (27.0)
Height, mean (SD), cm	161.0 (29.9)	165.6 (9.7)
Weight, mean (SD), kg	78.6 (24.1)	79.7 (19.2)
CHADS_2_ score, median (IQR)	2 (1-3)	2 (2-3)
Medical history		
Type 2 diabetes	9 (23.1)	9 (22.0)
Hypertension	30 (76.9)	33 (80.5)
Heart failure	13 (33.3)	15 (36.6)
Ischemic stroke or TIA	8 (20.5)	7 (17.1)
Systemic embolism	3 (7.7)	1 (2.4)
Coronary artery disease	8 (20.5)	13 (31.7)
Peripheral artery disease	0	2 (4.9)
Sleep apnea	5 (12.8)	6 (14.6)
Chronic kidney disease^c^	19 (48.7)	22 (55.0)
Cancer	6 (15.4)	8 (19.5)
Smoking		
Current	1 (2.6)	4 (9.8)
Past	15 (38.5)	14 (34.2)
Creatinine clearance, mean (SD), mL/min	57.6 (29.2)	56.8 (22.3)
Medications		
β-Blocker	27 (69.2)	27 (65.9)
Calcium channel blocker	13 (33.3)	16 (39.0)
Antiarrhythmic	2 (5.1)	1 (2.4)
ACE inhibitor or ARB	23 (59.0)	24 (58.5)
Digoxin	4 (10.3)	3 (7.3)
Diuretic	7 (17.9)	4 (9.8)
Lipid level–lowering drug	23 (59.0)	23 (56.1)
OAC	32 (82.1)	34 (82.9)
Warfarin	8 (20.5)	9 (22.0)
Apixaban	6 (15.4)	12 (29.3)
Rivaroxaban	14 (35.9)	11 (26.8)
Dabigatran etexilate mesylate	3 (7.7)	2 (4.9)
Edoxaban	1 (2.6)	0
Aspirin	4 (10.3)	5 (12.2)
Antiplatelet	0	1 (2.4)

Abbreviations: ACE, angiotensin-converting enzyme; ARB, angiotensin receptor blocker; BP, blood pressure; CHADS_2_, congestive heart failure, hypertension, age, diabetes, and stroke or transient ischemic attack; OAC, oral anticoagulant; TIA, transient ischemic attack.

^a^ Unless otherwise indicated, data are expressed as No. (%) of patients.

^b^ Includes First Nation, Inuit, or Métis; Latin American; South Asian; West Indian; not answered; or other.

^c^ One patient was missing in the control group.

### Guideline-Concordant OAC Use

Guideline-concordant OAC use at 3 months occurred in 36 of 39 patients (92.3%) in the early pharmacist intervention group vs 23 of 41 (56.1%) in the control group (*P* < .001), with an absolute increase of 34% and number needed to treat (NNT) of 3. Pharmacists received fax confirmation that all notifications were received by the PCP office. A fax notification is in keeping with the Alberta Pharmacists Standards of Practice for collaborative prescribing. During the 3-month follow-up period, 29 of 41 patients in the control group (70.7%) had 1 or more visits with their PCP. Notably, of the 23 patients in the control group who received appropriate OAC prescription, the PCP called the pharmacist for prescribing advice in 6 (26.1%). Among the 18 of 41 patients in the control group (43.9%) who were not receiving appropriate OAC therapy at 3 months, 7 continued into the delayed pharmacist intervention; 11 patients (61.1%) did not, as 4 declined, 2 no longer met the study inclusion criteria, and 5 were lost to follow-up. At 6 months (ie, 3 months after receiving the delayed pharmacist intervention), all 7 patients who went into the delayed intervention group were receiving guideline-concordant OAC therapy. Thus, 43 of the 46 patients exposed to the pharmacist intervention were receiving guideline concordant OAC therapy by 3 months. All patients in the early pharmacist intervention group and 21 of 23 (91.3%) in the control group were prescribed a direct OAC.

### Patient Satisfaction

Among the 42 patients in the pharmacist-led OAC intervention (early [n = 35] and delayed [n = 7]), 25 (59.5%) completed the survey. The median satisfaction score for each survey item is summarized in eTable 2 and eFigure 2 in Supplement 2. The median overall satisfaction score was 2.0 (IQR, 1.0-2.0). The median satisfaction score in the interpersonal relationship domain was 1.2 (IQR, 1.0-1.9) and in the quality-of-care domain was 1.6 (IQR, 1.0-2.0). The median score related to whether a pharmacist could improve was 3.0 (IQR, 3.0-3.0).

### Clinical Outcomes

During the 12-month follow-up period, 3 patients died (2 in the pharmacist intervention group and 1 in the control group), 1 had an ischemic stroke or transient ischemic attack (control group), and 5 bleeding events occurred (3 in the pharmacist intervention group [epistaxis managed medically, history of kidney transplant with hematuria, and progressive anemia found on outpatient laboratory evaluation] and 2 gastrointestinal tract bleeds in the control group). No patient had an acute coronary syndrome or a systemic embolism event. There was no significant difference between the pharmacist intervention and control groups in emergency department visits (13 vs 13; *P* = .88) or hospitalizations (4 vs 6; *P* = .55). At 1-year passive follow-up, OAC adherence was 32 of 35 (91.4%) in the pharmacist intervention group and 25 of 28 (89.3%) in the control group (*P* = .84).

## Discussion

To the best of our knowledge, this RCT is the first to demonstrate feasibility and efficacy of a pharmacist-led intervention to optimize OAC prescription for stroke risk reduction in patients with actionable AF presenting to community pharmacies. Patients in the pharmacist intervention group reported high satisfaction with pharmacists’ services. Rates of adverse clinical events were low, and there was no difference in use of health care services between the pharmacist-led intervention group compared with an enhanced usual care control group. Patients in both groups demonstrated high adherence to OAC therapy at 1 year.

Pharmacies offer an attractive setting for community-based AF screening.^53,54^ Screening for AF with pulse check and a smartphone-based ECG device in Australian pharmacies was found to be a feasible and cost-effective screening strategy^53^ and well-accepted among pharmacists and patients.^55^ In prior work, Sandhu et al^54^ enrolled individuals who were 65 years or older and were not taking OAC therapy into a stroke risk screening program that included an assessment for AF using a single-lead handheld ECG device, blood pressure measurements using an automated device, and diabetes risk calculated using a validated questionnaire over a 6-month period in Canadian community pharmacies. Screening results and recommendations for 1145 participants were provided to the primary care physician and among the 29 individuals (2.5%) identified as having actionable AF, only 5 (17.2%) had initiation of OAC therapy within 3 months.^54^ Although the findings of that study demonstrated a high prevalence of individuals who could benefit from stroke risk reduction therapy and that the intervention was cost-effective,^56^ the investigators highlighted the need for screening to be coupled with a defined care pathway (which we developed and evaluated in this study).

Pharmacies provide an ideal setting for addressing care gaps in OAC delivery and may be especially beneficial in underserved communities.^57,58,59^ In the US alone, there are 13 billion pharmacy visits per year, 270 million visits per week, and 4000 visits weekly per pharmacy. Nearly all US residents (91%) live within 5 miles of a community pharmacy.^60^ Pharmacists, who are specialized experts in medication-based therapies, are well positioned to assume a greater role in the management of chronic disease from overburdened PCPs. Whereas most patients may only see their PCP once a year, individuals visit community pharmacies at least twice and up to 8 times as frequently as their physician.^61,62^ This consistent interaction allows pharmacists to build trusted and longitudinal relationships.^63^ Accumulating evidence suggests that pharmacist-based interventions for managing chronic diseases in the community can be highly effective^28,64^ and sustainable^29^ and lead to greater adherence to guideline-directed targets and improved outcomes.^24,25,26,27,32,33,34,35,64^

This proof-of-concept work provides demonstration of efficacy of a pharmacist-based intervention for OAC prescription in a health care system where pharmacists can perform patient assessments; order and interpret laboratory results; initiate, adjust, and monitor drug therapy; access EHR and pharmacy databases; and work in a collaborative care model with physicians. Pharmacists took an average of 20 minutes to screen for eligibility, 30 minutes to perform a baseline visit, and 15 minutes for follow-up visits. Furthermore, we found low rates of adverse clinical events and high OAC therapy adherence in both groups. The high adherence in the control group may be explained by the OAC education received by the patients at time of study enrollment by the pharmacist, which is actually an enhancement over usual care for patients with AF filling prescriptions at their community pharmacy who are not receiving OAC therapy. Even with this existing infrastructure, implementation of our study revealed key learnings. First, stakeholder engagement prior to study implementation was important. There was hesitation on the part of some physicians who felt that pharmacists were potentially operating outside their scope of practice. To address this, the study protocol ensured pharmacists would communicate with the physician after each visit and that ultimately the PCP could override any OAC decisions. Second, despite overwhelmingly positive responses from pharmacies wanting to participate in the study, staffing issues, low prescription volumes, largely healthy and young clientele, and the COVID-19 pandemic led to lower than anticipated recruitment. Prescreening interviews to assess staff volumes, patient demographic characteristics, OAC prescription volumes, and prior research experience helped identify successful pharmacy sites. Last, limited knowledge about and/or comfort with managing OAC therapy was identified as a barrier in a prior survey evaluating the potential for pharmacists prescribing OAC therapy in AF.^65^ Therefore we created a comprehensive online training program including a knowledge assessment tool, in-person training, and a quality assurance program wherein a study coordinator who is a clinical pharmacist would randomly review screening logs of patients identified as ineligible and confirm inclusion criteria of all patients prior to enrollment.

### Limitations

This study has some limitations. The unit of randomization was the patient, which may have introduced contamination among enrollees from the same pharmacy. Future studies should consider alternative designs such as a cluster RCT at the level of the pharmacy to potentially minimize contamination. This study was conducted during the COVID-19 pandemic, which undoubtedly influenced screening volumes, the number of patients who were lost to follow-up, and response rates to the pharmacist satisfaction survey. There is potential for social desirability bias with the survey: the only patients responding were those who answered positively if asked about pharmacists. Further research on patient satisfaction in both the intervention and control groups is needed. We did not capture reasons from the PCP as to why a patient in the control group did not receive appropriate OAC therapy after notification. This information may further identify strategies for addressing care gaps. In addition to billing for services such as medication review, pharmacists were paid for participation (per patient, not per OAC prescription). Most patients enrolled in our study were White due to the demographics of north-central Alberta. A better understanding of how this intervention may work in diverse communities is needed. Scalability and sustainability of pharmacist OAC prescription will require larger trials demonstrating effectiveness and a clear health policy plan that expands pharmacist scope of practice and reimbursement for activities. A key element of our intervention was the ability of pharmacists to access a provincial EHR and to prescribe independently. Whether our findings are generalizable to other health care systems without pharmacists who can prescribe independently or through the use of collaborative practice agreements^41,42,43,44^ or pharmacists without access to full medical records requires further evaluation.

## Conclusions

In this RCT, we found pharmacist-led OAC prescription for patients with actionable AF resulted in significant and meaningful (absolute increase 34%, with an NNT of 3) increases in appropriate stroke risk reduction therapy for AF without an increase in adverse events or use of health care services. This study provides evidence of a potentially high-yield opportunity to effectively close gaps in the delivery of stroke risk reduction therapy for AF. Scalability and sustainability of pharmacist OAC prescription will require larger trials demonstrating effectiveness and safety.

## Notes

Supplement 1.
Trial Protocol

Supplement 2. **eFigure 1.** PIAAF Rx Study Trial Flow **eFigure 2.** Distribution of Responses for Pharmacist Satisfaction Survey **eTable 1.** Suboptimal Oral Anticoagulation Dosing **eTable 2.** Patient Satisfaction With Pharmacists Services Survey

Supplement 3.
Data Sharing Statement
