# Supplementary material for: Stroke Risk Reduction in Atrial Fibrillation Through Pharmacist Prescribing: A Randomized Clinical Trial
Source: JAMA Netw Open. 2024 Jul 24;7(7):e2421993. doi: 10.1001/jamanetworkopen.2024.21993 (PMC11270136; doi:10.1001/jamanetworkopen.2024.21993)
Supplement: Supplement 1. — Trial Protocol [file jamanetwopen-e2421993-s001.pdf]

I. The Need for a Trial

A. BACKGROUND

Atrial Fibrillation (AF) and Stroke Epidemiology

Atrial fibrillation (AF) is a leading cause of stroke, accounting for 15% of strokes overall and 25% of strokes in the elderly.[1] AF-related strokes are more severe, causing long-term physical and cognitive disability and are associated with 50% 1-year mortality.[1-3] Treatment for stroke is considerable, costing 3.6 billion dollars annually in Canada.[4] The potential burden of AF associated stroke is already a **critical health issue** which will only increase since the prevalence of AF and stroke attributable to AF increases substantially with age; more than 25% of Canadians over the age of 75 will develop AF.[5]

Oral anticoagulation (OAC) is a well-established therapy for stroke prevention. In a meta-analysis, warfarin was shown to reduce the risk of stroke 68% and mortality by 30% compared to placebo.[6] Recent randomized clinical trials have shown that compared to warfarin, new OAC therapies are easier to administer and reduce the risk of stroke/systemic embolism by 23%, total mortality by 11% and intracranial bleeding events by 54%.[7-10] Despite robust evidence OAC therapy is effective and safe; the availability of simple stroke risk stratification schemes and numerous guidelines providing strong recommendations regarding who would benefit from OAC therapy[11-13], less than two-thirds of eligible patients are prescribed OAC therapy.[14, 15] The public health consequence of undertreated AF are enormous, thus, alternative strategies to deliver stroke prevention therapy, such as pharmacist-directed interventions, need to be explored.

Expanding Scope of Pharmacist Practice

Pharmacist practices have been evolving over the last century, from managing medication dispensaries to managing drug therapy and prescribing. There are numerous studies demonstrating pharmacist involvement in disease monitoring and optimization of drug therapies for cardiovascular risk factors and heart failure increases adherence to guideline-based targets and improves outcomes.[16-20] In meta-analyses, pharmacist-directed care or pharmacist collaborative care with nurses or physicians were associated with significant reductions in systolic blood pressure[20] , low-density lipoprotein[18], improved hemoglobin A1c[19],and reduced heart failure hospitalizations[17] compared to usual care.

There are several reasons to explain the positive impact of pharmacist involvement: (1) pharmacists are highly accessible[21]; particularly important since physicians are managing a higher prevalence of chronic diseases resulting in patient health care demands that can often exceed their capacity to address them[22, 23]; (2) pharmacists have expertise in drug therapy (pharmacodynamics, pharmacokinetics, drug-drug interactions) allowing for effective and safe drug choices[24, 25];and (3) pharmacists are consistently ranked as the most trusted professionals[26] allowing them to establish important patient-provider relationships. Given these reasons and rising worldwide health care costs, several countries (United Kingdom, New Zealand, United States, Canada) have recently moved toward various forms of pharmacist independent or collaborative prescribing.[27-30]

Given traditional means of educating practitioners and patients on better risk factor control have limited effectiveness[31], a recent Cochrane review concluded that "nurse- or pharmacist-led care may be promising way forward…but these interventions require further evaluation."[32] In a recent randomized controlled study (PREVENTION led by Co-Investigator FA McAlister) among patients with previous stroke, pharmacist prescribing of anti-hypertensive and lipid lowering drugs led to significantly improved risk factor control compared to a nurse-led disease management control group.[33] In the PREVENTION study pharmacist with prescribing practices evaluated patients independently of their usual pharmacy procedures. In the setting of community pharmacies, pharmacist prescribing of antihypertensive medication alongside their usual clinical practice resulted in a clinically important and statistically significant reduction in blood pressure. Although, pharmacist-led anticoagulation clinics have been shown to improve time in therapeutic range of international normalized ratios, patient satisfaction and quality-of-life[17, 34-37], to our knowledge, no randomized trial has compared pharmacists initiation and/or optimization of OAC therapy compared to current practice models in AF patients for stroke prevention in a community setting.

B. Can we answer this question?

We believe we are uniquely placed to answer this question because Alberta was the first province in Canada to allow for additional prescribing authorization and as such pharmacists have the most experience in independent prescribing. According to the Alberta College of Pharmacists, there are 615 prescribing pharmacists to date, an increase of 98% from the prior year, and two-thirds practice in community settings.[38] Pharmacists have the ability to access electronic medical records, order lab tests and interpret results. Importantly, there is a strong track record of pharmacist-led intervention studies conducted by our research group in Alberta.[17, 18, 33-35, 39]

Summary:

Oral anticoagulation therapy for stroke prevention in AF is safe and effective but under-utilized. Optimal delivery of existing therapies would prevent AF-related stroke. In this study, we will first evaluate pharmacists' knowledge regarding appropriate use of OAC therapy in AF. We will then evaluate whether active pharmacist intervention could improve evidence-based use of OAC therapy in eligible AF patients. Lastly, we will evaluate the impact of active pharmacist intervention on healthcare utilization to help inform the economic analysis of pharmacist prescribing.

C. OBJECTIVES

Primary Objective:

To determine the difference in proportion of patients receiving optimal OAC therapy at 3 months in those randomized to intervention arm versus control arm.

Secondary Objectives:

(1) To determine the prevalence of patients with unrecognized AF eligible for OAC therapy and those with AF who should be on OAC therapy but are either not on or their existing OAC prescriptions; require adjustment due to contraindications, or sub-optimal levels (hereafter referred to those with "actionable" AF).
(2) To perform a qualitative review of implementation from pharmacists.

(3) To assess patient satisfaction with pharmacist services.
(4) To determine cost of the intervention.

D. METHODS

Study Design: This is a pilot prospective, open-label, randomized controlled trial with early versus late pharmacist intervention. The unit of randomization is the patient.

Study Setting: The study will be performed in community pharmacies with at least one pharmacist with independent prescribing authority as identified by the Alberta College of Pharmacists as successfully completed requirements and who have access to electronic medical records.

Study Population:

Participants will be eligible for <u>inclusion</u> if they meet the following:

1. Age $\geq$ 65 years with One additional stroke risk factor (hypertension, diabetes, heart failure - history of or left ventricular ejection fraction <0.40), previous stroke or transient ischemic attack)
2. AF and not on oral anticoagulation (OAC) therapy but eligible
3. AF on sub-optimal OAC
4. Written informed consent.

<u>Exclusion criteria</u>

1. AF on optimal OAC therapy
2. Inability to read or understand English
3. Participants considered unreliable by the Investigator or designated pharmacy team member concerning the requirements of follow-up, or with foreshortened life expectancy precluding 3 month follow-up
4. Severe cognitive impairment (≥5 errors on the Short Portable Mental Status Questionnaire)

We will identify those with 'actionable' AF (Table 1, Appendix) as those who are found to have AF (either based on single lead ECG or review of medical records), or those with known AF either not on OAC or require prescription adjustment for contraindications or sub-optimal OAC regimen.

Randomization: After obtaining informed consent, eligible participants with 'actionable' AF will be randomized to an early versus delayed pharmacist intervention arm using a central randomization number and to preserve concealed allocation.

Study Procedure (Figure 1, Appendix)**:**

(i) <u>Pre-Intervention:</u>

To aid in the implementation of the study, we will identify local community leaders, and organizations (pharmacists, PCP, local hospitals and community organizations) as part of a stakeholder team. This stakeholder team will meet to discuss study details and address potential barriers. Pharmacists with additional prescribing authority will be provided with training on AF management (symptoms, risk factors, AF-related outcomes, evidence-based guidelines on OAC therapy, bleeding risk) and a pharmacist who specializes in OAC management (T Bungard) will provide them with

comprehensive training on OAC management. Competency will be assessed after training. The educational seminars can be used to fulfill requirements for continued education.

Enrollment: Eligible participants will be recruited using direct advertisement and various in-store strategies (Table 2, Appendix).

The pre-intervention phase of the study will occur in two pharmacies with pharmacists who have additional prescribing authority in a single community and will be a 'run in' to the major clinical trial. The main objective of the pilot phase is to determine capacity and challenges prior to implementation of a larger clinical trial.
Specific Objectives of the pilot phase are:
1. To determine the number of patients recruited and screened
2. To determine time for screening and completion of case report form

RCT:

(ii) Intervention: Early versus Delayed (Figure 1):

Pharmacists will identify eligible participants as 65 years or older AND with one additional risk factor for stroke assessed by brief history and medical record review. The pharmacist will ask about a history of AF and confirm diagnosis by either electrocardiographic (ECG, Holter, event monitor) documentation or by physician documentation (notes or discharge summary). If AF is confirmed and the patient is taking oral anticoagulation appropriately (Table 1, Appendix) then they will be excluded from further participation. Stroke risk factors will also be confirmed based on medical record report or self-report and taking disease-specific medication (Table 3, Appendix).

For participants without a history of AF, the pharmacist will assess rhythm by using a mobile ECG apparatus attached to a smart phone or tablet, which will allow a single lead ECG recording (Alivecor) using two fingers on electrodes for 30 seconds. The single lead ECG recording includes a highly accurate and FDA-approved AF detector (sensitivity 98.5%, CI 92-100% and specificity 91.4%, CI 89-93%). Two ECG recordings will be obtained from each participant and if there is disagreement in the interpretation then a third ECG recording will be obtained. Real-time physician verification will be performed for ECG interpretations of AF. For patients with AF, a 12-lead ECG will be required within 24-48 hours of screening session however randomization will occur based on single lead ECG interpretation.

Participants with unrecognized AF who are eligible for OAC therapy (based on single lead ECG) and those with previously diagnosed AF who should be on OAC therapy but are either not on or their existing OAC prescriptions require adjustment due to, contraindications, or sub-optimal levels will be considered as having 'actionable' AF (Table 1, Appendix).

Participants with 'actionable' AF will be randomized (via a centralized secure website to ensure allocation concealment) in a 1: 1 ratio to either early versus delayed pharmacist intervention after obtaining informed consent.

The early intervention arm will have the following timeline:

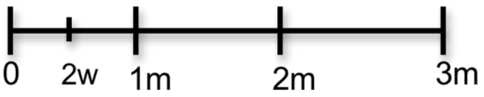

At time 0, participants will receive AF education, and pharmacists will complete baseline case report form, blood pressure assessment, prescribe OAC therapy or adjust existing prescription based on guideline algorithms; a summary of management will be kept for pharmacy records and a fax will be sent to the family physician regarding participation of the study. At 4 weeks, all participants taking oral anticoagulants will be seen in the pharmacy and the pharmacist/pharmacy technician will complete a follow-up checklist (Figure 4, Appendix).[40] If this is not possible, the participant will be contacted by telephone. As this is a pragmatic trial, additional follow-ups (in-person/phone) will be scheduled at the discretion of the pharmacist particularly if the patient had initiation of warfarin. The OAC clinic at the Mazankowski Heart Institute will provide support throughout this 3 month period if needed. Final follow-up and collection of close-out medications will occur at 3 months. Cardiologist support will be provided for any questions throughout the study period.

For patients randomized to the delayed intervention arm, during the first 3 months they will serve as a control group and will have a fax sent to their family physician identifying them as having ‘actionable’ AF and a medication list. The delayed intervention arm will follow the same procedures as the early intervention arm for those participants who did not have OAC optimized by their GP during the control phase. The delayed arm timeline will begin at day 90:

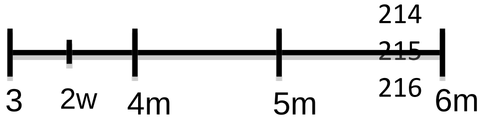

(iii) **<u>Follow-up</u>:** All participants will have follow-up after 3 months to assess current therapy by a blinded research staff. Follow-up will also entail review of pharmacy electronic records.

Primary Outcome: To determine the difference in proportion of patients receiving optimal OAC therapy at 3 months in those randomized to intervention arm versus control arm.
Optimal defined as a new prescription for OAC in a previously untreated AF or known AF who should be on OAC per guideline recommendations or adjustment of an existing OAC in a prescription.

Anticipated Results: The rate of new OAC prescriptions or appropriate changes to existing prescriptions will be higher in the intervention arm compared to the control arm.

Significance: This study will allow us to determine whether patients randomized to pharmacist intervention have a higher rate of appropriate OAC use compared to usual care. This may also provide early evidence for an expanded role for pharmacists, which includes prescribing of OAC therapy to address stroke prevention in AF.

Sample size and Statistical Analysis

Full trial

The primary goal of the full is to determine the rate of optimal OAC prescriptions in eligible participants between the active pharmacist arm and enhanced usual care arm. In a prior population-based study performed in Alberta[14], we found after an initial diagnosis of non-valvular AF 49% of patients were prescribed warfarin within 90 days. We do not have data for a baseline rate for prescribing OAC therapy by pharmacists. Therefore, we surveyed investigators from the C-SPIN to determine the clinically important change in OAC prescribing for an intervention. The consensus was an absolute minimal clinically important difference (MCID) of 15%. If we assume a MCID of 15% (absolute) higher in the intervention compared to control and 1:1 with 80% power at 5% alpha, we will require 168 participants per group. Assuming a dropout or loss to follow-up rate of at least 10%, the sample size will be a total of 370 patients.

All analyses were performed using an intention-to-treat principle of all randomized patients. Descriptive statistics will be used for comparing the two groups across the different outcomes.

Measurement of Secondary Outcomes:

A qualitative assessment of implementation by pharmacists will be performed with a brief interview using questions that were developed by the SEARCH-AF study.[41] Patient satisfaction with be assessed using the validated Patient Satisfaction with Pharmacists Services questionnaire 2.0.[42]

Healthcare Utilization

Healthcare utilization (hospitalization, emergency room visits, physician visits, use of laboratory testing, and use of OAC therapy) will be determined by interrogating patient electronic medical record (NETCARE) for study participants for up to 12 months after study enrollment. We will include in our consent form a specific section about accessing patient electronic medical records.

D. POSSIBLE PROBLEMS AND MITIGATING STRATEGIES

1) This is the first study to evaluate pharmacist prescribing for OAC therapy which may lead to hesitation or apprehension between pharmacists and primary care physicians. In order to overcome potential barriers, our stakeholder team will address potential issues through face-to-face meeting, teleconferences and email correspondence. Stakeholder team members will liaise with their respective communities regarding this study.

2) One of the challenges for any study is enrollment. We believe it is feasible to enroll our target sample size as prior Alberta pharmacist-based interventions have

successfully enrolled similar sample sizes. In addition, we will implement strategies based on experiences from the research team (RK Sandhu, J Healey, L Dolovich) conducting the Program for the Identification of Actionable Atrial Fibrillation – In the Pharmacy Setting (PIAAF-Pharmacy).

E. STUDY IMPORTANCE

No interventions for AF have been shown to improve survival and reduce serious morbidity beyond OAC therapy for stroke prevention. Effective and safe therapies exist to prevent AF associated stroke. This research will generate new knowledge on an innovative, and potentially sustainable stroke prevention strategy to increase evidence-based use of OAC therapy in patients with AF. These results will help to inform legislative and regulatory bodies in other provinces at various stages of expanding the scope of pharmacist practices and be a leader for other countries (UK, New Zealand, US) with existing independent or collaborative prescribing pharmacists.

II. TRIAL MANAGEMENT
STUDY ORGANIZATION

A. Central Coordinating Centre

The study will be coordinated by the SPOR/EPICORE center. EPICORE will be responsible for the electronic CRF, contracts, database and statistical support.

B ETHICAL STANDARDS

B.1 Ethical Considerations

Ethics Approval

This study will be conducted in compliance with the protocol, principles laid down in the Declaration of Helsinki, Good Clinical Practice (GCP), as defined by the International Conference on Harmonization (ICH), and ISO standards where applicable. Before study initiation, the Investigator must have written and dated approval/favorable opinion from the Institutional Review Board (IRB) for the protocol and consent form. Amendments to the protocol will also require IRB approval where applicable and in accordance with local laws and regulations.

Patient Consent

Prior to patient participation, written informed consent must be obtained from each patient (or legally accepted representative) and comply with the Declaration of Helsinki. The original signed consent must be retained on file by the Investigator and a copy given to the patient.

Risk Benefit Analysis

There are potential risks to patients participating in this study related to the use of OAC therapy including bleeding. An Ontario-based survey evaluating patient’s values and

preferences regarding relative importance of preventing stroke avoiding bleeding found patients were willing to endure 4.4 major bleeds in order to prevent one stroke.[43] In addition, we would be increasing appropriate use of OAC therapy for stroke prevention concordant with the AF guidelines leading to benefit for the participant of the study.

B.2. Patient Confidentiality

All patient information will be stored on a high security computer system and kept strictly confidential. Subject confidentiality will be further ensured by utilizing subject identification code numbers to correspond to outcome data in the computerized files.

Individual subject medical information obtained as a result of this study is considered confidential and disclosure to third parties is prohibited except for the following reason; Medical information may be given to the subject’s personal physician or to other appropriate medical personnel responsible for the subject’s welfare.

C DATA HANDLING AND RECORD KEEPING

Study personnel at participating sites will complete electronic case report forms (e-CRFs) through accessing a computerized program via the internet and entering the required trial information directly into the e-CRFs for electronic submission to a server located at VIGOUR. Consent forms will be filed at the Investigator’s site and made available for study related monitoring, audits, IRB review and any regulatory inspections if required. The Investigator must retain all study records in accordance with applicable regulatory requirements. A Data Management Plan will be developed by EPICORE and outline the detailed strategies to ensure quality in data collection and reporting.

APPENDIX

Table 1. Definitions for 'Actionable' Atrial Fibrillation (AF)

**Actionable AF (non-valvular)**	Unrecognized AF<br>Previously diagnosed AF not on oral anticoagulation (OAC) therapy but eligible<br>Previously diagnosed AF but require adjustment due to contraindication, or sub-optimal levels<br>- Warfarin : average INR not between 2-3 in last 2 months<br>- Rivaroxaban: not on 20 mg daily or 15 mg po daily for CrCL 30-49ml/min or patients with CrCL < 30ml/min taking any dose of rivaroxaban<br>- Dabigatran: not on 150 mg po bid or 110 mg po bid (in patients $\geq$ 75 years or CrCL 30-50ml/min) or patients with CrCL < 30ml/min taking any dose of dabigatran<br>- Apixaban: not on 5 mg bid or 2.5 mg bid (for patients with 2 of the following: age $\geq$ 80 years, body weight $\leq$ 60 kg or serum creatinine $\geq$ 133umol/L) or patients with CrCL < 25ml/min taking any dose of apixaban<br><br>**CrCl formula (for Cr measure in umol/L): CrCl=(140-age)(weight in kg) x 1.23 (x 0.85 if female)/SerumCr (umol/L)**
	For patients with a previous diagnosis of AF this should be confirmed by medical record report (notes, discharge summary, cardioversion) or electrographic evidence (ECG, holter, event monitor, echocardiogram)

Table 2. Recruitment Strategies

**Recruitment Strategies**	
Advertising	Local newspapers; senior community associations; senior events
In-store	Posters<br>Stand with an iPad near blood pressure kiosk with Alivcor technology for rhythm detection and prompt to see pharmacist<br>Verbal notification by pharmacist of patients who present for any prescription<br>Pharmacist/technician query database for age $\geq$ 65 years AND any of the following on medication review to send invitation to attend screening session:<br>i) anti-arrhythmics (ie amiodarone, sotalol, flecainide, propafenone, disopyramide)<br>ii) digoxin<br>iii) ca channel blocker (ie. verapamil, diltiazem,norvasc)<br>iv) b-blocker (ie metoprolol, bisprolol, carvedilol,atenolol)<br>v) ace-inhibitor (ie.lisinopril, ramapril)/angiotension receptor blocker (ie. cozaar, losartan, diovan)<br><br>vi) thiazide (hydrochlorthiazide), lasix, spironolactone<br>vii) any glucose lowering medication<br>biguanides (ie metformin)<br>sulfonylureas (ie glyburide, glipizide)<br>meglitinides (ie repaglinide)<br>thiazolidinediones (pioglitazone)<br>DPP-4 inhibitors (ie sitagliptin)<br>alpha-glucosidase inhibitors (ie acarbose, miglitol)<br>insulin

Table 3. Defining Stroke Risk Factors

**Hypertension**	Medical record report (ie notes, discharge summary) of a diagnosis of hypertension<br><br>Or self-report and taking at least one of the following class of drugs: ace-inhibitor (ie.lisinopril, ramapril);angiotension receptor blocker (ie. cozaar, losartan, diovan);thiazide (hydrochlorthiazide); ca channel blocker (ie. verapamil, diltiazem, norvasc);b-blocker (ie atenolol, metoprolol, bisprolol)
**Diabetes**	Medical record report (ie notes, discharge summary) of a diagnosis of diabetes<br><br>Or self-report and taking at least one of the following class of drugs: biguanides (ie metformin); sulfonylureas (ie glyburide, glipizide);meglitinides (ie repaglinide); thiazolidinediones (pioglitazone); DPP-4 inhibitors (ie sitagliptin); alpha-glucosidase inhibitors (ie acarbose, miglitol)
**Heart Failure**	Medical record report (ie notes, discharge summary) of a diagnosis of heart failure<br><br>Or Cardiac imaging with left ventricular ejection fraction $\leq$ 0.40<br><br>Or self-report and taking loop or thiazide diuretics
**Stroke or Transient Ischemic Attack**	Medical record report (ie notes, discharge summary) of a diagnosis of Stroke or Transient Ischemic Attack<br>Or Brain imaging (CT or MRI) with previous infarct

Figure 1.

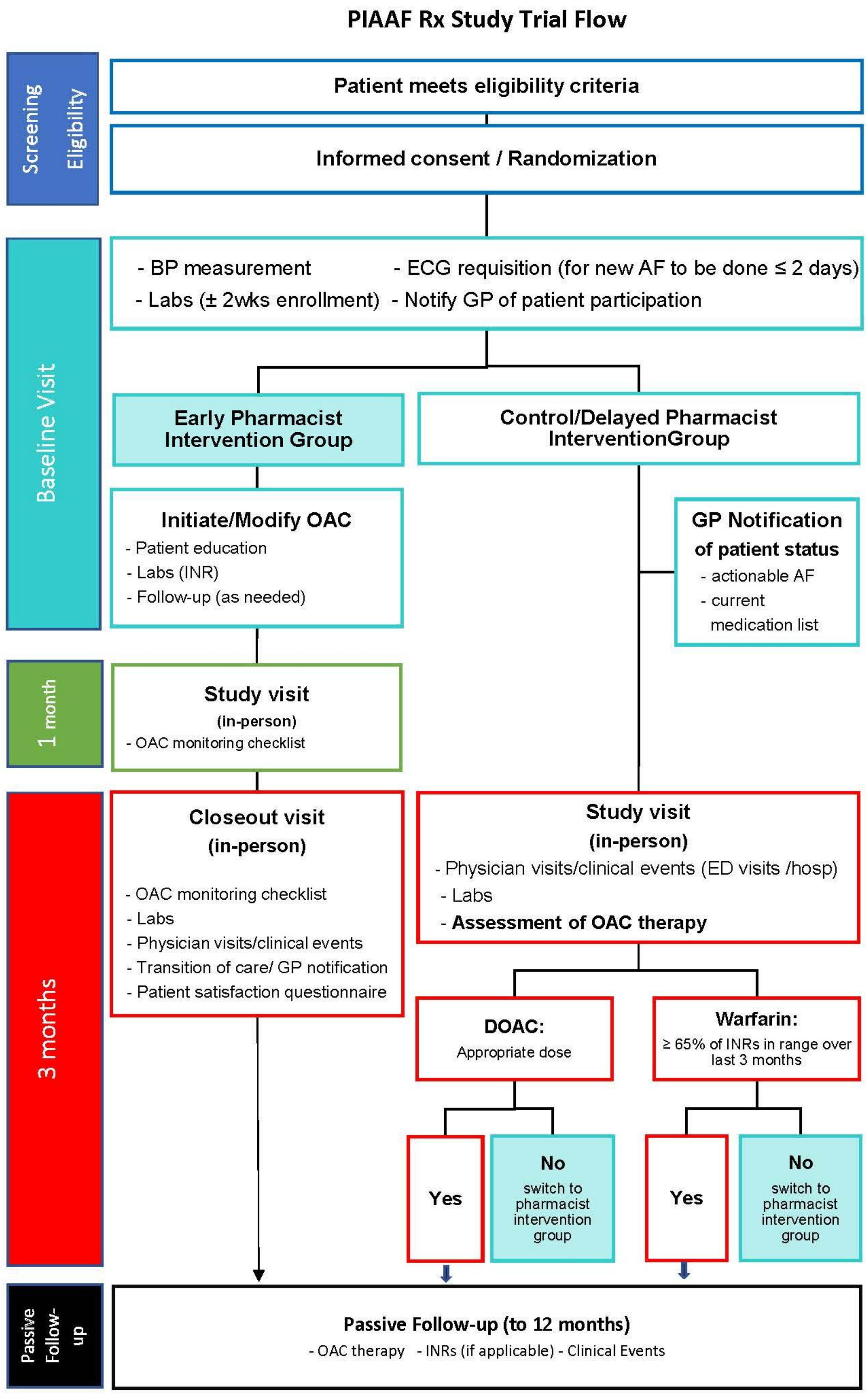
PIAAF Rx Study Trial Flow
Screening
Eligibility
Patient meets eligibility criteria
Informed consent / Randomization
Baseline Visit
- BP measurement
- ECG requisition (for new AF to be done ≤ 2 days)
- Labs (± 2wks enrollment)
- Notify GP of patient participation
Early Pharmacist Intervention Group
Control/Delayed Pharmacist InterventionGroup
Initiate/Modify OAC
- Patient education
- Labs (INR)
- Follow-up (as needed)
GP Notification of patient status
- actionable AF
- current medication list
1 month
Study visit
(in-person)
- OAC monitoring checklist
3 months
Closeout visit
(in-person)
- OAC monitoring checklist
- Labs
- Physician visits/clinical events
- Transition of care/ GP notification
- Patient satisfaction questionnaire
Study visit
(in-person)
- Physician visits/clinical events (ED visits /hosp)
- Labs
- Assessment of OAC therapy
DOAC:
Appropriate dose
Warfarin:
≥ 65% of INRs in range over last 3 months
Yes
No
switch to pharmacist intervention group
Yes
No
switch to pharmacist intervention group
Passive Follow-up
Passive Follow-up (to 12 months)
- OAC therapy - INRs (if applicable) - Clinical Events

380
381
