# Supplementary material for: Stroke Risk Reduction in Atrial Fibrillation Through Pharmacist Prescribing: A Randomized Clinical Trial
Source: JAMA Netw Open. 2024 Jul 24;7(7):e2421993. doi: 10.1001/jamanetworkopen.2024.21993 (PMC11270136; doi:10.1001/jamanetworkopen.2024.21993)
Supplement: Supplement 2. — eFigure 1. PIAAF Rx Study Trial Flow eFigure 2. Distribution of Responses for Pharmacist Satisfaction Survey eTable 1. Suboptimal Oral Anticoagulation Dosing eTable 2. Patient Satisfaction With Pharmacists Services Survey [file jamanetwopen-e2421993-s002.pdf]

Supplemental Online Content

Sandhu RK, Fradette M, Lin M, et al. Stroke risk reduction in atrial fibrillation through pharmacist prescribing: a randomized clinical trial. *JAMA Netw Open.* 2024;7(7):e2421993. doi:10.1001/jamanetworkopen.2024.21993

eFigure 1. PIAAF Rx Study Trial Flow
eFigure 2. Distribution of Responses for Pharmacist Satisfaction Survey
eTable 1. Suboptimal Oral Anticoagulation Dosing
eTable 2. Patient Satisfaction With Pharmacists Services Survey

This supplemental material has been provided by the authors to give readers additional information about their work.

© 2024 Sandhu RK et al. *JAMA Network Open.*

eFigure 1. PIAAF Rx Study Trial Flow

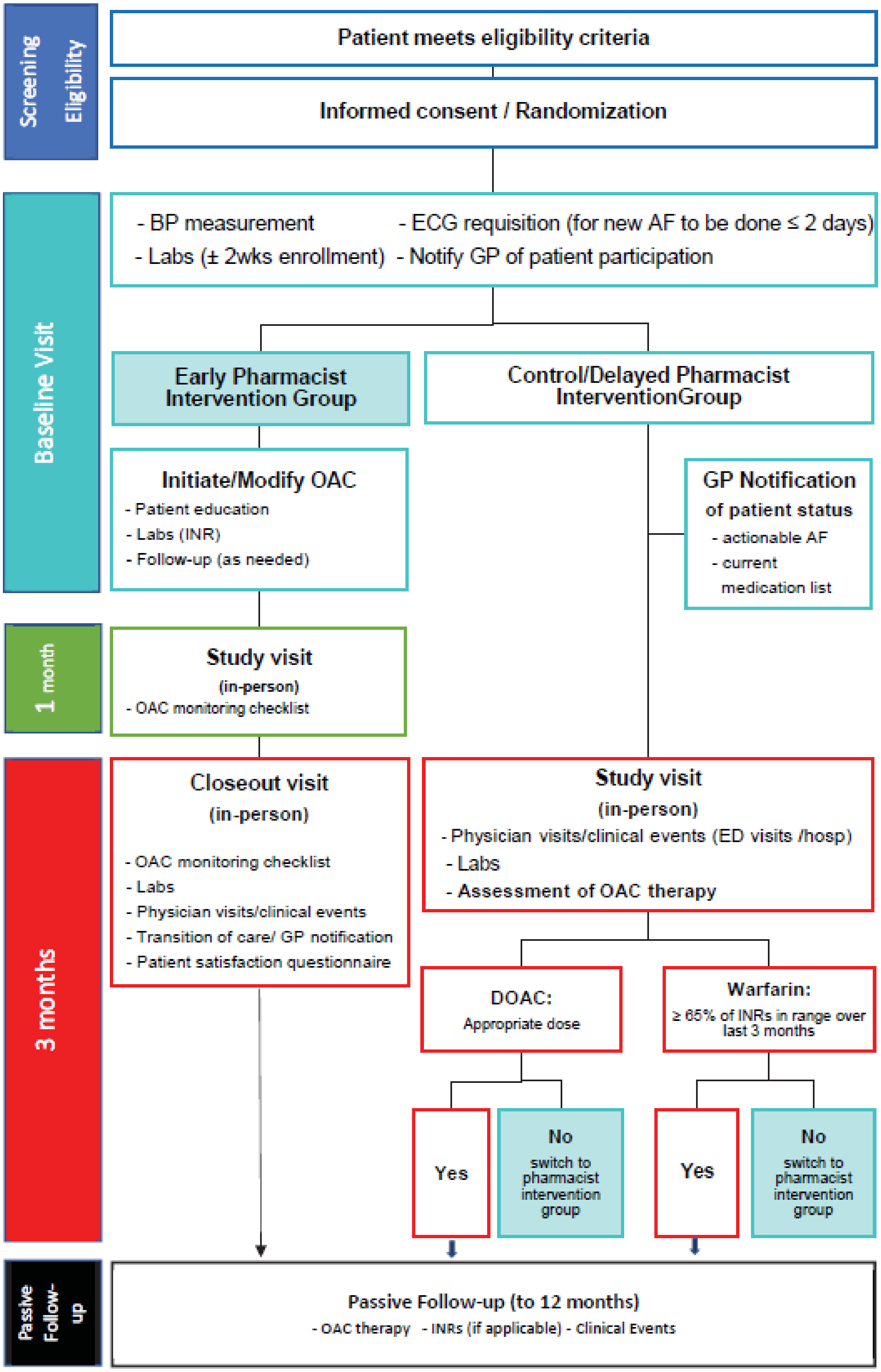

© 2024 Sandhu RK et al. *JAMA Network Open.*

eFigure 2. Distribution of Responses for Pharmacist Satisfaction Survey

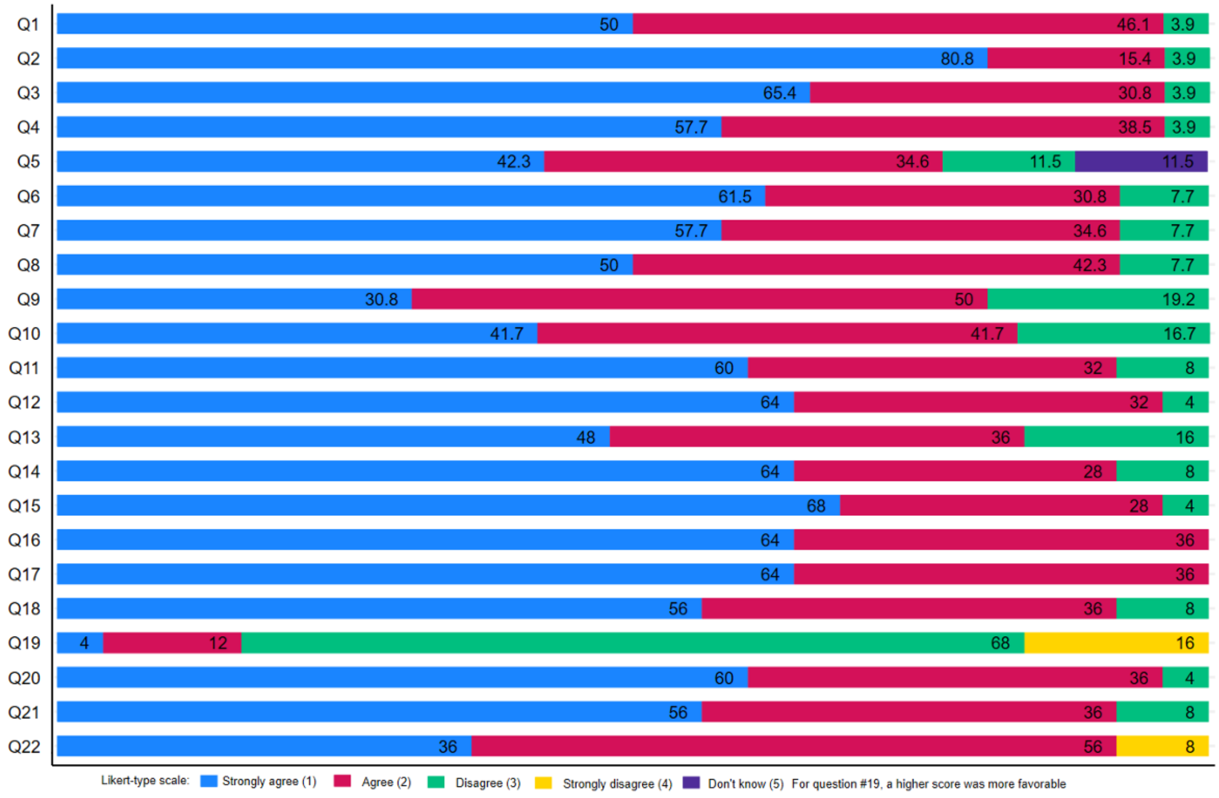

© 2024 Sandhu RK et al. *JAMA Network Open.*

eTable 1. Suboptimal Oral Anticoagulation Dosing

**Known Atrial Fibrillation and Sub-optimal dosing.**	- Warfarin : INR not between 2-3 in last 2 months<br>- Rivaroxaban: not on 20 mg daily, or not on 15 mg po daily for CrCL 30-49ml/min or taking any dose in patients with CrCL < 30ml/min<br>- Dabigatran: not on 150 mg po bid, or 110 mg po bid (in patients $\geq$ 80 years or those $\geq$ 75 years with at least one other bleeding risk factor or CrCL 30-50ml/min) or taking any dose in patients with CrCL < 30ml/min<br>- Apixaban: not on 5 mg bid or not on 2.5 mg bid for patients with 2 of 3 following criteria: age $\geq$ 80 years, body weight $\leq$ 60 kg or serum creatinine $\geq$ 133umol/L or taking in patients with CrCL < 25ml/min

© 2024 Sandhu RK et al. *JAMA Network Open.*

eTable 2. Patient Satisfaction With Pharmacists Services Survey

Question	Median Score (IQR)
1. Pharmacist fully addressed the main health reason/concerns/issues during my visit	1 (1-2)
2. The pharmacist was professional in all our interactions	1 (1-1)
3. The pharmacist explained information to me in a manner that I could understand	1 (1-2)
4. The pharmacist checked to see if I understood all the information	1 (1-2)
5. My physician and pharmacist worked together as a team to manage my health	2 (1-2)
6. The pharmacist spent as much time necessary to help me with my questions and concerns	1 (1-2)
7. The pharmacist made sure I understood how important it is to follow the drug regimen	1 (1-2)
8. The pharmacist provided useful recommendations on how to take my medications	1 (1-2)
9. The pharmacist provided useful recommendations about managing my overall health	2 (1-2)
10. The pharmacist worked with me to manage my medication related issues	2 (1-2)
11. The pharmacist followed up on my progress in a timely manner	1 (1-2)
12. The pharmacist was caring and kind in dealing with my health issues	1 (1-2)
13. The pharmacist encouraged me to achieve my treatment goals	2 (1-2)
14. I felt comfortable in my interactions with the pharmacist	1 (1-2)
15. The pharmacist was respectful to me during our interactions	1 (1-2)
16. The pharmacist was committed to improving my health	1 (1-2)
17. I could trust the information that the pharmacist provided	1 (1-2)
18. I was satisfied with the overall care provided by my pharmacist	1 (1-2)
19. There are some things about my visit with the pharmacist that can be improved	3 (3-3)
20. I would recommend my pharmacist to people I know	1 (1-2)

© 2024 Sandhu RK et al. *JAMA Network Open.*

21. If needed, I would continue seeing this pharmacist for my healthcare needs	1 (1-2)
22. The overall care provided by the pharmacist	2 (1-2)

Likert-type scale (strongly agree (1), agree (2) , disagree (3), strongly disagree (4)]. For question #19 '', a higher score was more favorable.

© 2024 Sandhu RK et al. *JAMA Network Open*.
