# Supplementary material for: Stroke Risk Reduction in Atrial Fibrillation Through Pharmacist Prescribing: A Randomized Clinical Trial
Source: JAMA Netw Open. 2024 Jul 24;7(7):e2421993. doi: 10.1001/jamanetworkopen.2024.21993 (PMC11270136; doi:10.1001/jamanetworkopen.2024.21993)
Supplement: Supplement 3. — Data Sharing Statement [file jamanetwopen-e2421993-s003.pdf]

Data Sharing Statement

Sandhu. Stroke Risk Reduction in Atrial Fibrillation Through Pharmacist Prescribing. *JAMA Netw Open*. Published July 24, 2024. doi:10.1001/jamanetworkopen.2024.21993

Data
Data available: No
